# Liposomal Amphotericin B and Leishmaniasis: Dose and Response

**DOI:** 10.4103/0974-777X.62886

**Published:** 2010

**Authors:** Shyam Sundar, Jaya Chakravarty

**Affiliations:** *Department of Medicine, Institute of Medical Sciences, Banaras Hindu University, Varanasi – 221 005, India*

**Keywords:** Kala-azar, Liposomal, Amphotericin B

## Abstract

Liposomal amphotericin B has been used with increasing frequency to treat visceral leishmaniasis (VL). It is the treatment of choice for immunocompetent patients in the Mediterranean region and the preferred drug for HIV/VL co-infection. Although there is a regional variation in the susceptibility of the parasite a total dose of 20 mg/kg is effective in immunocompetent patients. Randomized clinical trials of liposomal amphotericin B in the treatment and secondary prophylaxis of HIV-VL coinfected patients is urgently needed to optimize treatment in this subset. With the availability of Liposomal amphotericin B at a preferential pricing in the endemic areas, short course combination therapy can become a viable alternative.

*Leishmania donovani* is an intracellular protozoan parasite of genus *Leishmania*. It causes visceral leishmaniasis (VL), which is a disseminated and most serious form of leishmaniasis. The subgenus *L. donovani* complex consists of three species:*L. donovani*, the causative organism for VL in the Indian subcontinent and East Africa; *L. infantum*, which causes VL in the Mediterranean basin; and *L. chagasi*, which is responsible for the disease in Central and South America.

VL causes an estimated 500,000 new cases of disease and 60,000 deaths each year. Ninety percent of cases occur in: India, Bangladesh, Nepal, Sudan, and Brazil.[1] In South Asia and the Horn of Africa, the predominant mode of transmission is anthroponotic and humans with *kala-azar* or post-*kala-azar* dermal leishmaniasis provide the major reservoir for ongoing transmission.[2–4] In the Mediterranean, the Middle East, and Brazil, the disease is zoonotic, with the domestic dog as the most important reservoir host sustaining transmission.[2] In these regions, most human VL diseases occur in children or immunocompromised adults.

## PRESENT TREATMENT GUIDELINES

There is a regional variation in response to antileishmanial drugs and thus recommendations vary for treatment in different regions. Pentavalent antimonial compounds (Sb^v^) remain effective and preferred treatment in Africa, South America, Bangladesh, Nepal and India (except North Bihar) at the dose of 20 mg/kg/day parenterally for 28-30 days.

In North Bihar, where Sb^v^ resistance is high, the National Expert Committee has recommended amphotericin B deoxycholate to be used as the first line drug.[5] Due to inadequate infrastructure for the administration of amphotericin B this recommendation could not be implemented and the recommendations are currently undergoing transition. In the Mediterranean basin, the treatment of choice for immunocompetent patients is liposomal amphotericin B (L-AmB).[6] The drug of choice for the treatment of HIV/VL co-infection is an extended course of L-AmB in a total dose of 40 mg/kg.[7,8] Periodic L-AmB infusion has shown to prevent relapse in this subset of patients.[9]

## REVIEW OF ANTILEISHMANIAL AGENTS

The treatment of VL has many limitations. All antileishmanial drugs except miltefosine have to be given parenterally. The duration of therapy is long. Most of these drugs are toxic, need hospitalization and close monitoring making the treatment costly and beyond the reach of most patients. Rampant misuse of Sb^v^ led to the emergence of antimony resistance in India. The first report of resistance came in early 1980s, after which many modifications in dose and duration were made.[10–13] However, in 1997, only 36% patients could be cured by sodium stibogluconate in North Bihar at the dose of 20 mg/kg for 30 days.[14] Pentamidine was the first drug to be used in these antimony resistant patients, but its serious toxicities and declining efficacy led to discontinuation of its use.[15,16]

Due to the failure of existing drugs, amphotericin B was reintroduced for treatment of refractory VL in India.[17] Amphotericin B has excellent cure rate (~100%) at a dose of 0.75-1 mg/kg for 15-20 daily or alternate days intravenous infusions, however, most of the patients experience infusion reactions– eg, fever, chills, and thrombophlebitis–and occasionally serious toxicity–eg, hypokalemia, nephrotoxicity, myocarditis, and even death. These adverse effects necessitate close monitoring and hospitalization and ultimately increase the cost of therapy.

In lipid formulations of amphotericin B, deoxycholate is replaced with other lipids leading to less exposure of the free drug to organs. These formulations are based on the concept of targeted drug delivery to macrophages in the liver, spleen and bone marrow: the cells and organs affected in VL. Thus the tolerance is greatly improved and adverse effects including hypokalemia and nephrotoxicity are greatly reduced. By using these formulations it is possible to deliver larger doses of the drug over short periods of time. At present, three formulations have been tested extensively in VL: liposomal amphotericin B (AmBisome; Gilead Sciences), amphotericin B lipid complex (ABLC; Abelcet®, Enzon Pharmaceuticals), and amphotericin B cholesterol dispersion (ABCD; Amphotec™, InterMune Corp.).

Miltefosine, the first oral antileishmanial agent was registered for use in India in March 2002. At the dose of 50-100 mg for 28 days, the final cure rate was 94%.[18] Its limitation are high cost, need for monitoring for gastrointestinal side effects, occasional hepatic and nephrotoxicity. As it is teratogenic, women of child bearing potential have to observe contraception for the duration of treatment and an additional three months because of its half life of nearly one week. Further, its long half life also makes it vulnerable to rapid development of drug resistance.

Sitamaquine (WR-6026) is another orally administrable primaquine analogue which has completed phase II trials in India and Kenya. In India, at the dose of 1.75 and 2 mg/kg/day for 28 days the cure rates were 89% and 100% respectively.[19] In Kenya, with 2, 2.5 and 3 mg/Kg/day doses the cure rates were 80%, 82% and 91% respectively.[20] However, this drug is still at the developmental stage.

Paromomycin (PM) or aminosidine, a broad-spectrum aminoglycosidic aminocyclitols at a dose of 11 mg per kilogram of body weight intramuscularly daily for 21 days has a cure rate of 94.6% in India.[21] Major advantage of this drug is its cost, approximately US$ 10 - 15 for an adult patient and its reasonable safety profile.[22] However, requirement for injections for three weeks, monitoring of serum transaminase, lack of adequate data regarding its safety in pregnancy are its disadvantages.

The need of the hour is a drug which is efficient, safe, affordable with a shorter duration of therapy.

## LIPOSOMAL AMPHOTERICIN B

Liposomal amphotericin B (L-AmB) is a formulation of amphotericin B in which the drug is packaged with cholesterol and other phospholipids within a small unilamellar liposome. It is approved as an empirical therapy for presumed fungal infection in febrile neutropenic patients, treatment of cryptococcal meningitis in HIV-infected patients, treatment of patients with *Aspergillus* species, *Candida* species and/or *Cryptococcus* species infections refractory to amphotericin B deoxycholate, or in patients where renal impairment or unacceptable toxicity precluding the use of amphotericin B deoxycholate. The mechanism of leishmanicidal action is thought to be drug-binding to parasite ergosterol precursors, such as lanosterol, causing disruption of the parasite membrane.

## PHARMACOLOGY AND PHARMACOKINETICS OF LIPOSOMAL AMPHOTERICIN B

In comparison to amphotericin B deoxycholate, L-AmB produced higher plasma exposures and lower volumes of distribution and markedly decreased the excretion of unchanged drug in urine and feces.[23] The specialized formulation has characteristics that increase efficacy while minimizing toxicity: effective penetration and sustained levels in tissue, especially liver and spleen; high transition temperature leading to stability in blood, macrophages, and tissues; presence of cholesterol in the liposome, which minimizes drug interaction with mammalian cell membranes and decreases toxicity; and high affinity for ergosterol and its precursors ensuring antimicrobial efficacy.[24]

Higher initial doses (>5 mg/kg) provide better penetration and longer tissue persistence than do frequent low doses, suggesting that initial loading doses may increase efficacy. L-AmB has triphasic plasma profiles with long terminal half-life (152 ± 116 h), but plasma concentrations were higher (*P<*0.01) after administration of L-AmB (maximum concentration of drug in serum [*C*max], 22.9 ± _ 10 μg/ml) than those of Amphotericin B deoxycholate (AB) (*C*max, 1.4 ± _ 0.2 μg/ml). Although liposomal amphotericin B was administered at a dose 3.3-fold higher than that of AB, plasma concentrations during the first 24 h were 8- to 16-fold higher in L-AmB-treated subjects. Renal and fecal clearances of L-AmB were 10-fold lower than those of AB (*P<*0.01). The ability of liposomes to sequester drugs in circulating liposomes and within deep tissue compartments may account for these differences.[23]

Significant accumulations of amphotericin B into reticuloendothelial organs have been observed, with 239 ± 39 μg/g found in the liver after chronic LAmB dosing (5 mg/kg/day), which was seven times higher than the 33 ± 6 μg/g after AB dosing (1 mg/kg/day) (*P*=0.002). Accumulation in kidneys, however, remained 14-fold lower (*P=* 0.04) following LAmB dosing (0.87 ± 0.61 μg/g) than after AB dosing (12.7 ± 4.6, μg/g).[25] Although transient increases in the creatinine level can occur, acute and chronic toxicity is uncommon even with doses up to 15 mg/kg.[26]

## CLINICAL TRIALS OF LIPOSOMAL AMPHOTERICIN B FOR VL

In the Indian subcontinent, different regimens of L- AmB have been tested with the objective to find the lowest total dose with acceptable efficacy. In one of the first studies with L- AmB, 30 parasitologically confirmed patients were randomly divided into three equal treatment groups; Group 1 received L- AmB 2mg/kg on days 1-6, and 10 (total dose 14 mg/kg); Group 2 received L- AmB 2 mg/kg on days 1-4, and 10 (total dose 10 mg/kg); Group 3 received the same dosage on 1, 5 and 10 (total dose 6 mg/kg)[Table 1]. All the patient showed 100% cure rate.[27] In another study, single dose of 15mg/kg of AmBisome in 17 patients showed 100% cure rate and was much better tolerated than amphotericin B.[28] Around the same time, low dose L- AmB (5 mg/kg), given either as a five-day course or as a single infusion, gave a cure rate of 93% and 91% respectively.[29] Encouraged by the efficacy of low doses of L- AmB a dose-ranging, multicenter trial was conducted, consisting of 84 patients with visceral leishmaniasis refractory to antimony therapy. L- AmB was administered at cumulative doses of 3.75, 7.5, and 15.0 mg/kg for five consecutive days, which resulted in definite cure rates of 89, 93, and 97%, respectively suggesting that low-dose L- AmB can cure most patients with Indian *kala-azar*.[30] In a fairly large multicenter trial (n=203), a single dose of 7.5 mg/kg yielded a 90% cure rate at 6 months.[31] Studies from the Indian subcontinent also suggested that liposomal amphotericin B caused substantially lower rates of toxicity than conventional amphotericin B deoxycholate or amphotericin B lipid complex (ABLC).[28,32] Based on the above trials WHO recommended that 15 or 10 mg/kg of L-AmB may be adequate to achieve high cure rates in Southern Asia.[33]

**Table 1:** Efficacy and toxicity of various dosing regimens of liposomal amphotericin B (LAmB) in immunocompetent patients with visceral leishmaniasis

Country	Reference(s)	Study design	No. of subjects		Total AmB dose, mg/kg	LAmB regimen	Percentage of cured subjects	Follow-up duration, months	Reported adverse events^a^

			Total	Per group					
Brazil	35^b^	Open-label, dose-finding	32	15	20	2 mg/kg on day 1–10	87	6	Fever, 41%; chills, 9%; respiratory distress, 6%; cardiac arrhythmia, 9%; treatment was stopped for 2 subjects
				4	10	2 mg/kg on days 1–4 and 10	100	6	
				13	14	1–2 mg/kg on days 1–6 and 10	62	6	
Greece	38	Open-label with historical control	123^c^	41	20	10 mg/kg on days 1–2	98	6	Fever and chills, 7%; no discontinuations of treatment
				30	20	4 mg/kg on days 1–5	90	6	
Italy	8	Open-label, dose-finding	31^d^	10	30	3 mg/kg on days 1–10	100	12–24	Nonsignificant in BUN level; no change in creatinine level; no discontinuations of treatment
				10	21	1–1.4 mg/kg on days 1–21	100	12–24	
Italy^e^	36	Open-label, dose-finding	88^f^	32	15	3 mg/kg on days 1–4 and 10	91	12	Mild adverse effects; increase in BUN and creatinine levels; no dis- continuations of treatment
				42	18	3 mg/kg on days 1–5 and 10	98	12
				13	24	4 mg/kg on days 1–5 and 10	100	12	
Italy	37	Open-label, dose-finding	106^c^	16	15	3 mg/kg on days 1–3, 5, and 10	75	12	No adverse events, no change in levels of BUN, creatinine, electrolytes, or liver enzymes
				66	18	3 mg/kg on days 1–5 and 10	98	12	-
				11	21	1 mg/kg on days 1–21	100	12	-
				13	30	3 mg/kg on days 1–10	100	12	-
India	27,35^b^	Open-label, dose-finding	30	10	6	2 mg/kg on days 1, 5, and 10	100	6	One patient had fever, and 2 had chills; no discontinuations of treatment
				10	10	2 mg/kg on days 1-4 and 10	100	6	-
				10	14	1-2 mg/kg on days 1-6 and 10	100	6	-
India	28	Randomized, open-label equivalency	34	17	15	Single 15-mg/kg dose	100	6	Chills, 17% (65% of subjects in ConAMB group); nausea, 6% (53% of subjects in ConAmB group)
India	29	Open-label, dose-finding	91	46	5	Single 5-mg/kg dose	91	6	Fever and/or chills, 49%; vomiting, 4%; back pai, 2%; no change in creatinine level
				45	5	1 mg/kg on days 1-5	93	6	-
India	30	Randomized, double blind, dose-finding	84^g^	28	3.75	0.75 mg/kg on days 1-5	89	6	Infusion-related rigors, 44%; fever, 36%; back pain, 10%; transient increase in creatinine level, 8%
				28	7.5	1.5 mg/kg on days 1-5	93	6	-
				28	15	3 mg/kg on days 1-5	97	6	-
India	31	Open-label non-comparison	203	203	7.5	Single 7.5 mg/kg dose	90	6	Fever, 10%; Chills, 3%; vomiting, 4%; back pain, 2%; no renal toxicity
India	32	Randomized, open-label equivalency	153	51	10	2 mg/kg on days 1-5	96	6	Fever, 29%; rigors in 98% of subjects in ConAmB group; no increase in creatinine level (but a significant ConAmB group)
Kenya	35^b^	Open-label, dose-finding	25	5	6	2 mg/kg on days 1, 5, and 10	20	6	Few
				10	10	2 mg/kg on days 1-4 and 10	90	6	-
				10	14	1-2 mg/kg on days 1-6 and 10	100	6	-
Sudan	34	Open-label, dose-finding	49	16	12	3-5 mg/kg on days 1, 3, and 10	50	Passive	Clinical evaluation only; 4 instances of extravasation; patients in study were severely ill
				16	24	3-5 mg/kg on days 1, 2, 6, 8, 10, and 13	88	Passive	-

NOTE. BUN, blood urea nitrogen; ConAmB, conventional AmB desoxycholate.

a) Incidence of adverse events in the LAmB group (versus comparison group, where appropriate)

b) Multicenter trial in Brazil, India, and Kenya,

c) All subjects were children,

d) Study population included 15 immunocompetent children, 5 immunocompetent adults, and 11 immunocompromised adults,

e) Study included 83 cases from Italy, three cases from Brazil, and two cases treated in the United Kingdom,

f) Study population included 56 children and 32 adults,

g) Patients who did not respond to or relapsed after treatment with pentavalent antimonial drugs

In Africa, clinical trial data with L- AmB are very few. In 1995 an open label trial for L- AmB was conducted in Sudan for the treatment of complicated visceral leishmaniasis. Patients selected for the trial had either relapsed after treatment with a combination of pentavalent antimony (Sb^v^) and aminosidine or had incomplete parasitological response to Sb^v^ and aminosidine, or had severe illness. Drug regimen 1 (3 doses of 3-5 mg/kg) cured 8 (50%) of 16 patients; regimen 2 (6 doses of 3-5 mg/kg) cured 14 (88%) of 16. The optimal regimen of L-AmB in this study was administration of 4 mg/kg on days 0, 3, 6, 8, 10, and 13.[34] In a phase II clinical trial in India, Brazil and Kenya the efficacy and safety of liposomal amphotericin B was evaluated, first cohort received 2 mg/kg/day (MKD) on days 1-6 and day 10 (total dose, 14 mg/kg), the second cohort received 2 MKD on days 1-4 and day 10 (total dose, 10 mg/kg) and a third cohort was administered 2 MKD on days 1, 5, and 10 (total dose, 6 mg/kg). In Kenya, the first regimen cured all 10 patients (100%), the second cured 9 of 10 patients (90%), and the third cured only 1 of 5 patients (20%).[35]

In Europe, a variety of regimens of liposomal amphotericin B have been tried. Most of these clinical trials demonstrated 90%–98% efficacy with a total dose of 18–21 mg/kg in immunocompetent patients. In a multi-centre study in Mediterranean basin *(Leishmania infantum*), ten patients (six children) received L-AmB at the dose of 1–1.38 mg/kg/day for 21 days, and 10 (nine children) received 3 mg/kg/day for 10 days. All were cured without significant adverse events and without relapse during 12–24 months of follow-up.[8] In another study, from this region, of 88 immunocompetent patients (56 children) different doses of L- AmB were given. Thirteen patients received a total dose, 24 mg/kg, and all were cured; 42 received a total dose of 18 mg/kg and 41 were cured; 32 received a total dose15 mg/kg, and 29 were cured. One adult was cured with a total dose of 12mg/kg. The authors recommended a total dose of AmBisome of ≥ 20 mg/kg, given in five or more doses of 3-4 mg/kg over ≥ 10 days.[36] In a study of 106 immunocompetent children, the authors concluded that the optimal regimen in immunocompetent children with L. infantum visceral leishmaniasis would be a total dose of 18 mg/kg of L- AmB, 3 mg/kg per day for five days, followed by 3 mg/kg administered as an outpatient regimen on day 10.[37] In an open prospective study from Greece, forty-one children with parasitologically confirmed leishmaniasis received L-AmB, 10 mg/kg daily for two days who were compared to 30 children who, in a previous study, were treated with L-AmB, 4 mg/kg daily for five days. At six months after completion of treatment, 40 of 41 children treated with two doses of L-AmB were cured as compared to 27 of 30 children treated with 5 doses of L-AmB.[38] Many pediatricians currently use a regimen of 10 mg/kg/day of L-AmB for 2 consecutive days.[39] For imported cases in the United States, the US Food and Drug Administration recommends 3 mg/kg on days 1-5, 14, and 21, for a total dose of 21 mg/kg.[40] In New Zealand, the recommended regimen is 1–1.5 mg/kg for 21 days or 3 mg/kg for 10 days.

## LIPOSOMAL AMPHOTERICIN B IN HIV-VL COINFECTION

There have been no formal randomized clinical trials of liposomal amphotericin B treatment or secondary prophylaxis regimens in HIV-VL coinfected patients. Most of the data available are from case series[41–43] [Table 2]. The absence of a specific T cell response against *Leishmania* in HIV-infected patients prevents its elimination and the persistence of *Leishmania* does not allow reconstitution of the patients’ immune status. This negative interaction between the two infections leads to a high rate of recurrence. Thus, secondary prophylaxis seems to be mandatory. The efficacy of Sb^V^ and L- AmB were comparable in most case studies, but the lower rate of toxicity for L- AmB has caused most clinicians to consider it to be the antileishmanial drug of choice in VL-HIV-coinfected patients.

**Table 2:** Findings of published studies of liposomal amphotericin B (LAmB) treatment in HIV-visceral leishmaniasis-coinfected patients

Country	Reference	Study design	No. of subjects	Total LAmB dose, mg/kg	Regimen	Initial response	Relapse rate, %
Spain	41	Case series (relapse after Sb^v^ treatment)	2	22.5	1-1.5 mg/kg per day for 15 days	Good clinical response, parasite free at 3-6 months	0
				21	1 mg/kg per day for 21 days	-	-
Greece	42	Case series, secondary prophylaxis	2	40	1 mg/kg per day for days 1-7 and 1.5 mg/kg per day for days 8-29, followed by 1 mg/kg or 50 mg twice a month	Good clinical response; no relapse at 10-16 months	0
				20	0.75 mg/kg per day for days 1-7 and 1.5 mg/kg per day for days 8-17 followed by 1 mg/kg or 100 mg twice a month	-	-
Spain	43	Case series	5	40	4 mg/kg per day for days 1-5, 10, 17, 31, and 38	Parasites cleared in 80% of subjects	40^a^
Europe^b^	8	Open-label, dose-finding	11	29-39	100 mg per day for 21 days	Negative for parasites at day 21; 8 of 11 subjects relapsed in 3-22 months	89^c^
Italy	7	Open-label, dose finding	10	40	4 mg/kg per day for days 1-5, 10, 17,24, 31, and 38	Negative for parasites at day 45; 7 out of 8 subjects relapsed at 2-7 months	88^d^
France	9	Case series, secondary prophylaxis	5	60-86 by day 30	2.9-4.1 mg/kg per day for 5-24 days, followed by 2.7-3.8 mg/kg every 15 days to prevent relapse	3 of 5 subjects were relapse free at months 13-22	40^e^

NOTE. SB^V^, pentavalent antimonial drugs.

a) Relapses at 4 and 20 months.

b) Nine subjects from Italy, 1 from France, and 1 from Portugal.

c) Two deaths due to other causes, 8 relapses, and 1 cure.

d) Seven subjects experienced relapses at 2–7 months, 2 were lost to follow-up, and 1 was listed as “leishmanina positive”.

e) Two patients had relapse at 42 and 270 days and were re-treated with high-dose liposomal LAmB followed by prophylaxis, with good response in 1 of the 2 patients

In a study by Davidson *et al.* seven patients were treated with 100mg L-AmB/day for 21 days (giving a total dose of 29-38.9mg/kg); the five patients who showed initial parasitological recovery all had post-treatment relapses.[8]

In an attempt to reduce the number of relapses, Russo *et al*. used high and intermittent doses (4mg/kg.day on days 1-5, 10,17, 24, 32 and 39) to treat 10 HIV-positive patients. This regimen appeared similar in efficacy to the lower dosage used by the previous study and did not manage to prevent relapses.[7] In a case series of five patients in France, L-AmB at a dose of 2.9-4.1mg/kg per for 5-24 days, followed by 2.7-3.8mg/kg every 15 days to prevent relapse decreased the relapse rate to 40%.[9]

In a study from Spain, 17 HIV patients, with at least one previous episode of VL who received L-AmB as secondary prophylaxis for VL, were included to measure the proportion of patients remaining free (non-relapse) of VL at different time points. Each VL episode was treated with four mg/kg/day of L-AmB intravenously for five consecutive days and once per week thereafter for five more weeks (total, 10 doses=40 mg/kg). Once cure had been determined, all patients received 5 mg/kg of intravenous L-AmB every 3 weeks as secondary prophylaxis. The probability of remaining free of relapse at 6 months was 89.7% (95% CI, 76.2–100); at 12 months, the probability was 79.1% (95% CI, 61–97.2) and at 24 and 36 months, the probability was 55.9% (95% CI, 30.5–81.3). In the non-relapsing group, patients had a significant increase in CD4 cell levels, whereas in the relapsing group, no significant increase was observed.[44] Secondary prophylaxis with doses of liposomal amphotericin B or other antileishmanials every two to four weeks after initial clinical cure of VL is now the standard of care in Europe,[45–47] but data are insufficient to recommend a specific regimen.

## LIPOSOMAL AMPHOTERICIN B IN COMBINATION THERAPY

With increasing efforts to improve the treatment of visceral leishmaniasis, there is a growing interest in combination therapy, as practiced in the treatment of tuberculosis, HIV infection, and malaria. Such an approach, in the form of Sb^v^ plus aminosidine (paromomycin), was tested initially in Kenya, India, and Sudan in the 1990s which showed enhanced overall efficacy and/or reduced treatment duration.[48–50] The potential advantages of two-drug chemotherapy in the treatment of VL are less toxicity (as a result of lower drug doses and/or shorter treatment courses); convenience, better compliance, and lower costs, resulting from less lengthy treatment; and possibly reduced likelihood of developing resistance to either agent.

Recently, a randomized, noncomparative, group-sequential, triangular design study assigned 181 subjects to treatment with 5 mg/kg of L-AmB alone (Group A; 45 subjects), 5 mg/kg of L-AmB followed by miltefosine for 10 days (Group B; 46 subjects) or 14 days (Group C; 45 subjects), or 3.75 mg/kg of L-AmB followed by miltefosine for 14 days (Group D; 45 subjects). When it became apparent that all regimens were effective, 45 additional, nonrandomized patients were assigned to receive 5 mg/kg of L-AmB followed by miltefosine for seven days (group E). All 226 subjects showed initial apparent cure responses. Nine months after treatment, final cure rates were similar: group A, 91% (95% confidence interval [CI], 78%–97%]; group B, 98% (95% CI, 87%–100%); group C, 96% (95% CI, 84%–99%]; group D, 96% (95% CI, 84%–99%); and group E, 98% (95% CI, 87%–100%).

These results suggest that single infusion of L-AmB (in most instances, administered in an outpatient setting) followed by a brief self-administered course of miltefosine could be an excellent option against Indian *kala-azar*.[51] At present a number of trials are going on to evaluate different combination treatment regimens (co-Administration), of AmBisome, Paromomycin and Miltefosine in India. (NCT00523965, NCT00371995)

## LIPOSOMAL AMPHOTERICIN B PRICING

In 1992, an agreement between the WHO and Vestar led to preferential pricing for liposomal amphotericin B for patients with VL of $50 (in US dollars) per vial; a negotiation in 2004 led to the even more reduced price of €22.30 per vial. This price was valid for liposomal amphotericin B for patients with VL who are treated by not-for-profit institutions in East Africa. However, a preferential pricing agreement with WHO (agreement between Gilead and WHO of 14 March 2007) has recently reduced the price of L-AmB (AmBisome®) for endemic regions to $20 per 50-mg vial.[52] Even with preferential pricing, liposomal amphotericin B (total dose, 20 mg/kg) is not as cost-effective as other first-line regimens (i.e., Sb^V^, paromomycin, and conventional amphotericin B). However, preferential pricing increases the prospect of combining L-AmB in combination regimens.

## CONCLUSION

In zoonotic VL (the Mediterranean Basin, the Middle East, and Brazil) a total liposomal amphotericin B dose of ≥20 mg/kg is adequate to treat immunocompetent children and adults in these regions. The exact dosing schedule can be flexible (divided into doses of 10 mg/kg on 2 consecutive days or in smaller divided doses), but liposomal amphotericin B pharmacokinetics suggest that the initial dose will provide better tissue levels if at least 5 mg/kg is given. The schedule of 10 mg/kg/day on two consecutive days needs to be validated in adults.

For HIV-VL coinfection, Highly Active Antiretroviral Therapy should be a priority. There is an urgent need for multicenter trials of L-AmB as a first-line treatment and for secondary prophylaxis of VL in HIV-infected patients. In the anthroponotic cycle in the Horn of Africa, liposomal amphotericin B can be given at a total dose of 20 mg/kg however, a dose of 10–15 mg/kg may be adequate for South Asia.

In India, where a single infusion of L-AmB by itself at 7.5 or 5 mg/kg can induce cure rates of 90%–91%, combination regimen with lower doses of L-AmB with miltefosine or paromomycin is an option. With the preferential pricing, along with just one day of hospitalization, makes a single infusion of 10 mg/kg of L-AmB considerably less expensive and a viable option for the treatment of VL in the subcontinent.[53]. Well conducted trials of combination therapy with L-AmB is urgently needed.
